# Diagnostic and Therapeutic Value of Endoscopic Retrograde Cholangiopancreatography in Acute Cholangitis: A Study From Kashmir Valley, India

**DOI:** 10.7759/cureus.74396

**Published:** 2024-11-25

**Authors:** Basharat Ahmed Ganie, Saleem Javaid Wani, Uksim Qadri, Mushtaq Khan, Nazir Ahmad Dar

**Affiliations:** 1 Internal Medicine, Sher-i-Kashmir Institute of Medical Sciences, Srinagar, IND; 2 Infectious Disease, Sher-i-Kashmir Institute of Medical Sciences, Srinagar, IND; 3 Gastroenterology, Sher-i-Kashmir Institute of Medical Sciences, Srinagar, IND; 4 Radiation Oncology, Sher-i-Kashmir Institute of Medical Sciences, Srinagar, IND

**Keywords:** acute cholangitis, benign, bile duct infection, endoscopic retrograde cholangiopancreatography (ercp), malignant

## Abstract

Background

Cholangitis, or bile duct infection, can present in two primary forms, namely, acute ascending cholangitis (the milder form) and acute fulminant cholangitis (the more severe variety). In all types of cholangitis, bile duct obstruction occurs, with choledocholithiasis (the presence of gallstones in the bile duct) being the leading cause of this blockage. *Escherichia coli* is the most commonly isolated pathogen in these infections. If the biliary duct infection becomes suppurative, sepsis is a common complication. Cholangitis can lead to significant morbidity and mortality, which may persist even with prompt treatment or drainage. Endoscopic retrograde cholangiopancreatography (ERCP) remains a crucial diagnostic and therapeutic tool for managing acute cholangitis. Despite its importance in managing cholangitis, there is limited research on the characteristics and outcomes of ERCP in patients with acute cholangitis from Kashmir in northern India. This study was conducted to explore the role and effectiveness of ERCP in the diagnosis and treatment of acute cholangitis.

Methodology

This observational study was conducted in the Department of Gastroenterology at a tertiary care hospital in North India over a period of two years, from January 1, 2018, to December 31, 2019. The study included 48 consecutive patients diagnosed with acute cholangitis who underwent ERCP for both diagnostic and therapeutic purposes.

Results

The median age of the patients included in the study was 55 years and the male-to-female ratio in the study cohort was 1.6:1, with 30 (62.5%) males and 18 (37.5%) females. Among the patients with comorbid conditions, hypertension was the most common, present in 12.5% of cases. Choledocholithiasis was the most frequent diagnosis, identified in 70.83% of patients. Ultrasonography of the abdomen revealed choledocholithiasis in 34 (70.83%) cases, the most common radiological finding. A comparison of clinical symptoms and diagnostic test results before and after 72 hours of ERCP demonstrated a statistically significant improvement in patient outcomes following the procedure.

Conclusions

In contrast to other diagnostic approaches for evaluating biliary duct obstruction, timely ERCP remains a reliable and effective option for both diagnosis and therapeutic intervention in acute cholangitis. It is associated with improved diagnostic accuracy, enhanced therapeutic outcomes, and reduced morbidity and mortality rates, making it a critical tool in managing this potentially life-threatening condition.

## Introduction

Acute cholangitis, or ascending cholangitis, is a severe and potentially fatal condition resulting from a bacterial infection that ascends the biliary tree [1]. Charcot delineated the tripartite presentation of cholangitis in 1877, comprising right upper quadrant pain, fever, and jaundice, epitomized as Charcot’s triad. Concomitant biliary obstruction and the superimposition of bacterial colonization are classical cholangitis manifestations. Among clinical types, ascending cholangitis denotes the mildest form, whereas acute fulminant cholangitis represents the most serious condition. Every manifestation of cholangitis is inextricably linked to biliary impediment, albeit not all biliary occlusions precipitate cholangitis [2-5]. Reynolds and Dragon augmented Charcot’s triad with septicemia and cognitive impairment in 1959, formulating the quintessential “Reynolds pentad” [6,7].

Choledocholithiasis is the most common antecedent of biliary occlusion, whereas neoplastic entities, benign strictures, and interventions upon the bile ducts may also instigate such obstruction. Ascending infection or portal bacteremia frequently underlie biliary duct contamination, heralding the clinical onset of cholangitis [8]. The pivotal determinant of morbidity is “choledochal pressure.” Beyond a threshold of 25 cm H_2_O, hepatic immunological competence falters, and the defense mechanisms against infection get disrupted [8]. Pathophysiologically, cholangitis manifests via biliary duct obstruction, intraluminal pressure escalation, and bile contamination [9]. In 25-40% of cases, choledochal pressures surpassing 25 cm H_2_O correlate with infection dissemination into intrahepatic canaliculi, cholangiovenous reflux, and ingress into the hepatic vasculature and lymphatic conduits, culminating in bacteremia. Suppurative infections frequently culminate in sepsis [10]. Antecedent to therapeutic innovations encompassing endoscopic retrograde cholangiopancreatography (ERCP) and potent antimicrobial agents, the mortality rate for this clinical constellation, previously at 80-90%, has plummeted to a mere 5-15% [8]. However, in the presence of concomitant medical morbidities and advanced age, mortality rates range between 17% and 40%. Noteworthy reduction in mortality is achieved through judicious endoscopic drainage post-stabilization of patients [11].

## Materials and methods

The study was conducted in the Department of Gastroenterology of a tertiary care hospital in northern India over a two-year period, from January 1, 2018, to December 31, 2019.

Inclusion and exclusion criteria

Patients diagnosed with acute cholangitis for the first time were included in the study. The sample size was determined using Cohen’s formula. Patients with a prior history of hospitalization due to cholangitis or those whose hospital stay exceeded 24 hours were excluded from the study.

Study population

A total of 48 consecutive patients diagnosed with acute cholangitis and who underwent ERCP during the study period were enrolled. Written informed consent was obtained from all participants following an approved study protocol.

Methodology

Data Collection

A detailed review of the patients’ medical histories and thorough clinical examinations were performed.

Blood Sampling

Blood samples were collected before ERCP and within 72 hours post-procedure for biochemical and microbiological analysis.

Tumor Markers

In cases of diagnostic uncertainty or in elderly patients, tumor markers were also evaluated.

Imaging Studies

For additional diagnostic support, transabdominal ultrasonography (USG) was performed. If required, computed tomography (CT) or magnetic resonance imaging (MRI) was conducted.

The diagnosis of acute cholangitis was made in accordance with the following Tokyo Guidelines (2018) [12]: (A) Systemic inflammation: A-1: fever and/or shaking chills. A-2: Laboratory data: evidence of inflammatory response (abnormal white blood cell counts, increased serum C-reactive protein levels, and other changes indicating inflammation). (B) Cholestasis: B-1: Jaundice: total bilirubin more than or equal to 2 mg/dL. B-2: Laboratory data (abnormal liver function tests, including increased serum alkaline phosphatase, gamma-glutamyltransferase, aspartate aminotransferase, and alanine aminotransferase levels). (C) Imaging: C-1: Biliary dilatation. C-2: Evidence of the etiology on imaging (stricture, stone, stent, etc.).

The diagnosis was suspected with one item in A and one item in either B or C. The diagnosis was definite with one item in A, one item in B, and one item in C.

Procedures were performed using Pentax side-view duodenoscope, and 10 cm biliary stents of 8.5 or 10 F diameter. According to the culture sensitivity, antibiotics were administered both before the procedure and after the procedure until patients were afebrile and hemodynamically stable and fit for discharge.

Statistical analysis

Data analysis was done on an MS Windows-based computer. The data were first keyed into a Microsoft Excel spreadsheet and cleaned for any inaccuracies. Statistical analysis was done using SPSS Statistics for Windows Version 27.0 (IBM Corp., Armonk, NY, USA). Categorical variables were shown in the form of frequencies and percentages. The t-test was performed to assess the significance of laboratory parameters before and after 72 hours of ERCP.

Ethical clearance

The study was conducted following the Institutional Ethics Committee guidelines and the Helsinki Declaration of 1964, revised in 2013. This study was approved by the Institutional Ethics Committee of Sher-i-Kashmir Institute of Medical Sciences (protocol number: RP-17/2019 dated 16-02-2019).

## Results

The obstruction of the biliary ducts, precipitating an elevation in intraluminal pressure and consequent infection of the bile, constitutes a cardinal aspect in the pathophysiology of acute cholangitis. Unquestionably, each bout of cholangitis is underpinned by biliary duct obstruction. The study included a cohort of 48 individuals, comprising 30 (62.5%) males and 18 (37.5%) females. The mean age of the patients was 54 years and the age range was 35-80 years. Hypertension was the prevailing comorbidity, afflicting six (12.5%) patients, whereas 32 patients exhibited no underlying pathologies. Jaundice (87.5%) emerged as the predominant clinical manifestation, followed by pyrexia (77.1%), abdominal distress (75%), and hypotension, with altered sensorium, which was assessed using the Glasgow Coma Scale, manifesting in 10.4% of instances. The various reasons causing obstruction of the biliary ducts, characterized by purulent cholangitis diagnosis according to the Tokyo criteria, are delineated in Table 1. Choledocholithiasis was the most frequent diagnosis, afflicting approximately 34 (70.83%) patients, while biliary ascariasis was the least common, affecting two (4.17%) patients, as presented in Table 1.

**Table 1:** Primary diseases found in the study population.

Disease	n = 48	%
Choledocholithiasis	34	70.83
Cholangiocarcinoma	4	8.33
Pancreatic carcinoma	4	8.33
Biliary stricture (due to previous laparoscopic cholecystectomy)	4	8.33
Biliary ascariasis	2	4.17

Clinical findings and laboratory parameters were significantly relieved following ERCP (Tables 2, 3). No complications were noted in the study population.

**Table 2:** Clinical findings of the study population.

Finding	Before (n)	%	After (n)	%
Jaundice	42	87.5	2	4.17
Pain (especially in the right upper quadrant)	36	75.0	3	6.25
Fever	37	77.1	7	14.58
Hypotension, disturbed consciousness	5	10.4	0	0.00

**Table 3:** Laboratory parameters of patients before the procedure and 72 hours after the procedure. *: P-value obtained using the t-test. ALT: alanine aminotransferase; ALP: alkaline phosphatase

Laboratory values	Before the procedure	72 hours after the procedure	P-value
	Mean ± SD	Mean ± SD	
Total bilirubin (mg/dL)	8.73 ± 6.16	4.99 ± 5.05	0.001*
ALT (U/L)	160 ± 108.74	106.83 ±74.06	0.001*
ALP (U/L)	425.75 ± 182.01	314.04 ± 147.34	0.001*
Albumin (g/dL)	3.31 ± 0.868	3.15 ± 0.887	0.101

The bile culture was positive in 28 (58.3%) of the 48 patients included in the study. The most frequently isolated organisms were gram-negative bacteria, including *Escherichia coli* in 17 (60.7%) of 28 positive bile cultures, *Pseudomonas aeruginosa* in nine (32.1%) of 28 positive bile cultures, and *Klebsiella pneumoniae* in two (7.1%) of 28 positive bile cultures (Table 4). These organisms were mainly sensitive to carbapenems, fluoroquinolones, and cephalosporins.

**Table 4:** Organisms isolated from the study cohort.

Organism isolated	Number	%
Escherichia coli	17	60.7
Pseudomonas aeruginosa	9	32.1
Klebsiella pneumoniae	2	7.1

## Discussion

Cholangitis is a serious infection of the bile ducts that leads to significant illness and death. It varies in severity, from the relatively mild acute ascending cholangitis to the severe acute fulminant cholangitis. The condition is caused by obstruction of the bile ducts, which allows infections to develop. The most common bacterial pathogens include *Escherichia coli* (27%), *Klebsiella* spp. (16%), *Enterococcus* spp. (15%), *Streptococcus* spp. (8%), *Enterobacter* spp. (7%), and *Pseudomonas* spp. (7%) [4,5,13]. Our study confirmed these findings, with *Escherichia coli* being the most commonly isolated bacteria, in agreement with the findings of Sokal et al. [14].

Under normal conditions, the pressure of bile secretion from the liver ranges between 120 and 150 cmH_2_O, while pressure in the extrahepatic bile ducts is between 100 and 150 cmH_2_O. The gallbladder receives bile at a pressure of 12 to 18 cmH2O, regulated by the movement of the sphincter of Oddi. If bile pressure exceeds 300 cmH_2_O, bile secretion from the liver stops. Similarly, when choledochal pressure exceeds 25 cmH_2_O, the liver’s ability to fight infections is compromised [8]. For patients, particularly those who are elderly or have other medical conditions, timely biliary drainage is crucial. Delays in drainage are associated with mortality rates between 17% and 40%, while elective drainage after stabilization reduces mortality to about 3% [15,16].

The severity of cholangitis can range widely, with some cases progressing to sepsis [5-7,17]. Diagnosing cholangitis can be challenging, as septic patients may be misdiagnosed up to 25% of the time [5,17]. For elderly patients presenting with abdominal pain and jaundice, it is essential to maintain a high degree of suspicion. Fever, reported in 90% of cholangitis cases in the literature, was seen in 77.1% of our patients. Abdominal pain in the upper right quadrant was present in 75% of our cases, close to the 80% reported in the literature. Jaundice was observed in 87.5% of our cases, higher than the 60% typically reported, likely due to our institution’s status as a referral center. Hypotension and confusion were observed in 10.4% of cases in our study compared to 15%-30% in existing reports [8].

Elevated levels of alkaline phosphatase were noted in 70% of patients, consistent with the 78% rate in the literature [8]. Bilirubin, C-reactive protein, and erythrocyte sedimentation rates were high in most cases. Evaluating calcium levels is essential for identifying pancreatitis, while electrolytes provide insights into kidney function. Coagulation profiles should also be checked, especially in patients with cirrhosis or disseminated intravascular coagulation, to guide further treatment. Tumor markers are particularly useful in elderly patients.

Diagnostic imaging played a significant role in our study. All patients underwent transabdominal USG, with CT or magnetic resonance cholangiopancreatography (MRCP) used as needed. USG revealed stones in the common bile duct in 70.83% of cases but showed choledochal dilation in 70% of cases, while stones were detected in only 13% of cases. This highlights the limitations of USG in definitively ruling out cholangitis. MRCP, a non-invasive diagnostic method, has an 85% sensitivity and 93% specificity for detecting gallstones and bile duct diseases. However, its sensitivity is lower for detecting small stones (<6 mm) or intrahepatic stones [17]. In our study, MRCP detected bile duct abnormalities in 70.83% of cases. CT scans, used in 20 patients, identified bile duct stones in 40% of cases, but they remain valuable for uncovering other causes and complications of cholangitis.

Limitations

The study’s findings cannot be broadly generalized due to the small sample size. Larger studies involving a broader population are needed to validate these results.

## Conclusions

Urgent biliary decompression is imperative in patients refractory to pharmacological intervention, presenting with septic manifestations and jaundice indicative of potential biliary obstruction and cholangitis. This study corroborates the efficacy of ERCP for biliary drainage. Post-procedural assessments demonstrated substantial amelioration of clinical and biochemical parameters. In summary, for acute suppurative cholangitis, prompt endoscopic interventions remain a robust approach, offering superior diagnostic and therapeutic efficacy while significantly mitigating morbidity and mortality compared to alternative biliary duct evaluation methodologies.
